# Does the sequence of plyometric and dynamic stretching exercises influence subsequent sprint performance? A randomized crossover intervention study

**DOI:** 10.5114/biolsport.2024.129485

**Published:** 2023-09-21

**Authors:** Devisson S. Silva, Daniel Boullosa, Erika V. M. Pereira, Micael D.J. Alves, Matheus S.S. Fernandes, Felipe J. Aidar, Leila F. dos Santos, Raphael F. de Souza

**Affiliations:** 1 Department of Physical Education, Federal University of Sergipe (UFS), São Cristóvão, Brazil; 2 Graduate Program in Physical Education, Federal University of Sergipe (UFS), São Cristóvão, Brazil; 3 Federal University of Mato Grosso do Sul, Campo Grande, Brazil; 4 Faculty of Physical Activity and Sports Sciences, Universidad de León, León, Spain; 5 Graduate Program in Neuropsychiatry and Behavioral Sciences, Federal University of Pernambuco (UFPE), Recife, Brazil

**Keywords:** Track and field, Athletic performance, PAPE, Muscle stretching exercises, Plyometric, Warm-up

## Abstract

The objectives of this study were to evaluate the acute effects of the sequence order of drop jumps (DJ) and dynamic stretching (DS) on sprinting performances in competitive athletes and to investigate the relationships between post-activation performance enhancement (PAPE) in sprint performance and lower limb power. Thirteen male jumpers and sprinters participated in this study (19 ± 2 years; 177 ± 7 cm; 71.7 ± 5.6 kg). Through a randomized crossover design, the athletes were exposed to three different conditions after a standardized warm-up: DS+DJ, DJ+DS, and control. Sprinting performance over 40 m was analysed with consideration of initial (0 to 20 m) and final acceleration (20 to 40 m) phases. The effect of intervention was examined by two-way repeated-measures of ANOVA. Pearson’s correlation test was used to determine the association between PAPE during sprinting and jump performance. There was no effect of any factor on 40-m sprint performance. Meanwhile, the performance at 20–40 m was higher after the DS+DJ condition when compared to baseline (8.79 ± 0.43 vs. 8.91 ± 0.35 m/s; p = 0.015). However, the initial acceleration was worsened in the DJ+DS condition when compared to baseline (6.26 ± 0.25 vs. 6.22 ± 0.26 m/s; p = 0.002). There was a negative correlation between CMJ height and the improvement in final acceleration (r = -0.741; p = 0.004). The use of DS prior to DJ is an effective strategy to improve performance in the final acceleration phase (20–40 m). The athletes with lower levels of lower limb power benefited the most from this PAPE strategy.

## INTRODUCTION

Warming up is an important part of athletes’ routine during both training sessions and competitions, aiming to provide adequate biomechanical and physiological conditions that can influence the performance of the subsequent main activity [1]. Hence, previous investigations have sought to determine the best warm-up strategies in various sports to promote higher levels of force production [2, 3], decrease the oxygen deficit [4], increase body temperature [1, 4] and induce post-activation performance enhancement (PAPE) [5, 6].

To promote PAPE, various types of conditioning activities (CAs) have been used, including traditional strength exercises [7], eccentric loading exercises [8], ballistic exercises [9] and sled towing [10], with the possibility of combining these exercises in different sequences [11]. However, some of these activities require the use of additional equipment, including supports, bars, etc., thus making them difficult to use in a competitive environment. From this perspective, the use of ballistic exercises can be considered a viable alternative without the need for complementary equipment [9, 12, 13], therefore presenting greater applicability in competitive settings. Ballistic exercises have also shown greater concentric speed, strength and muscle activation compared to a similar traditional exercise movement [14]. Among the most commonly used ballistic exercises are plyometric exercises (PE) [10, 12, 15] and dynamic stretching (DS) [16, 17].

The use of DS exercises during the warm-up has previously shown improvements in jumping performance [18–20], peak strength [19], and linear sprinting speed [19]. In addition, PAPE has also been observed in sprint [21, 22], countermovement jump (CMJ) [23] and competitive performances in athletics [24, 25] after implementation of PE in the warm-up routine. Interestingly, the inclusion of three depth jumps after DS promoted higher performance enhancements in a 20-m sprint when compared to DS alone [22]. Moreover, the inclusion of five DJs after DS promoted better performance in a 1000-m run in male runners [12]. In contrast, another previous study observed a better vertical jump performance after using ten minutes of DS when compared to the protocol that added three sets with three tuck jumps after DS [19]. Meanwhile, to date, there are no studies that have examined the use of DS after PE as a PAPE strategy. This information would be useful to determine whether the positive effects of these exercises on neuromuscular function and subsequent performance are additive or not depending on the exercise sequence used.

There is no consensus on the best combination or sequence of these exercises as a PAPE strategy. Meanwhile, other moderators such as strength and power levels may also influence PAPE effects [26] and should also be explored. Therefore, the aim of the current study was to evaluate the acute effects of exercise sequence (DS+DJ vs. DJ+DS) on sprint performance and determine whether the PAPE responses are related to lower limb power. We hypothesized that the combination of these stimuli could improve sprint performance after both protocols, and that athletes with the greater lower limb power would exhibit greater PAPE responses.

## MATERIALS AND METHODS

### Study design

A randomized crossover design was used to study the influence of DJ combined with DS as a strategy to enhance sprinting in track and field athletes. Our study was conducted at an athletics outdoor track over a 3-week period. Every subject was informed about the purpose, procedures, and risks of the study. All procedures were approved by the research ethics committee of the UFS (process number 4.890.323) and were carried out according to the Helsinki Declaration.

**FIG. 1:**
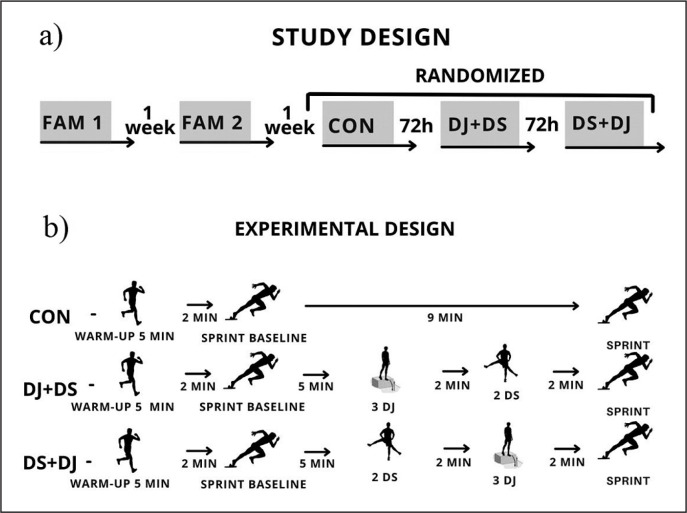
a) Study design; b) Experimental design FAM = Familiarization; CON = Control; DJ+DS= Plyometrics + dynamic stretching; DS+DJ = Dynamic stretching + plyometrics.

The subjects visited the athletics track on five different occasions: two familiarization sessions and three experimental sessions, 72 hours apart, thus allowing for an appropriate recovery period (see Figure 1). In the first familiarization session, the subjects performed the countermovement jump (CMJ) assessments, in addition to the warm-up protocols and the tests to be performed. In the second familiarization session, the optimal height for the drop jump was determined and the procedures of the first session were repeated, to minimize the learning effects for the subsequent experimental sessions. For the next experimental sessions, the subjects were randomly assigned, using Microsoft Office Excel software, to the three warm-up conditions: Dynamic Stretching + Drop Jump (DS+DJ), Drop Jumps + Dynamic Stretching (DJ+DS), and Control (C).

In each experimental session, the warm-up was performed after the baseline sprint, followed by the evaluation of the 40-m sprint performance with consideration of the 0–20-m (initial acceleration) and 20–40-m (final acceleration phase) phases. Subjects were recommended to maintain their eating and training routines throughout the experimental period, except for the 24 hours before the experimental sessions in which a resting day was recommended. The ingestion of alcohol and caffeine was not allowed before the experimental sessions.

### Subjects

The sample was composed of competitive male sprint (100- and 200-m dash) and jump (triple and long jump) athletes, who participated in the under-20 and adult championships in the State of Sergipe (Brazil). All the athletes who won medals in these specific events were eligible to participate in the research. The sample size was calculated a priori based on a statistical power of 0.95, assuming an effect size of 0.66 according to the findings of Lima et al. [27] and an alpha level of P < 0.05. The minimum sample size of 9 subjects was obtained (G*Power software package [version 3.1.9.4], Franz, Universitat Kiel, Germany). After sending invitations to all the eligible athletes, the following inclusion criteria were used for those who accepted to participate: (1) age of 16 years or older, (2) minimum of three training sessions per week, (3) minimum experience of six months in plyometric training, and (4) absence of lower limb injuries in the last six months. Sixteen athletes were finally included in the study. All subjects were encouraged to continue their normal training routines, except 24 hours before the experimental sessions. Exclusion criteria were suffering injuries during the study period and not attending any session of the study. All subjects signed an informed consent form. The parents of the athletes under the age of 18 signed the informed consent form.

### Drop jump assessment

During the second familiarization session, subjects completed a test to determine the optimal DJ height to be used in the next experimental sessions. Subjects performed three DJ from two different heights (20 and 40 cm) [28]. The optimal height was selected using the best reactive strength index (jump height/contact time). The jump was recorded and analysed using the My Jump 2 app [28], which was installed on an iPhone (v. 8, Apple Inc., Cupertino, CA, USA).

### Standard warm-up

In all conditions, the athletes completed a standardized 5-min warm-up which consisted of: 2-min × 500 m of slow running on the track, 4 running technique drills over 20 m with 20 s of active recovery by walking (totalizing 2:30 min); and a 5-m maximum acceleration. Two minutes after completing this warm-up protocol, the subjects performed the baseline sprint in all conditions.

### Dynamic stretching and drop jump protocols

The subjects performed two dynamic stretching exercises (vertical and lateral leg swings), 1 set of 15 repetitions for each leg, increasing the speed progressively until the 10^th^ repetition while maintaining the maximum amplitude and speed until the last repetition. The athletes performed three DJ with the optimal DJ height previously determined in the familiarization session. During the DJ, the subjects were instructed to keep their hands on their waist and perform the jumps as fast and high as possible.

### Sprint assessment

Subjects performed two 40-m linear sprints on the track, one after the standardized warm-up which served as baseline and another sprint after the potentiation protocols or control condition. All attempts were recorded using an iPhone 8 (Apple Inc., Cupertino, CA, USA) and the MySprint app (T-Mobile Inc., Bellevue, WA, USA). The system is based on high-speed video analysis (240 fps) and has been demonstrated to be valid and reliable for assessing linear sprint performance [29]. The athletes started from a three-stand position with their preferred hand on the track. The start of the sprint was determined when the athletes’ thumb took off after visual inspection. Six cones were located at 5, 10, 15, 20, 30, and 40 m to ensure that split times were recorded correctly. Two independent observers were asked to select the first frame in which the subjects’ right thumb left the ground (i.e., the start of the sprint) and the frames in which the marker placed on the pelvis was aligned with the cones for each sprint [29]. For data analysis, we used the mean velocities recorded in the 40-m sprint, and from 0 to 20 m (i.e. acceleration phase) and 20 to 40 m (i.e. final acceleration phase).

### Countermovement jump assessment

The evaluation of the CMJ was performed during the second familiarization session as a measure of lower limb power. CMJ performance was verified with the jump height (cm), which was estimated using a validated contact mat [30] (Chronojump, Bosco systems, Barcelona, Spain) following the flight-time method. The subject started in a standing upright position with his feet on a mat and with his hands on his hips, followed by a downward movement flexing his knees to approximately 90° to immediately jump as high as possible. Each athlete made three attempts separated by 10 s. The highest jump was used for further analyses.

### Statistical analysis

The results were presented as mean ± SD. The normality assumption for each variable was verified using the Shapiro-Wilk test. The effect of intervention on sprinting was examined by two-way repeated-measures ANOVA (3 conditions × 2 time points [baseline and post-CA]). Mauchly’s test of sphericity was applied, and the Greenhouse-Geisser Epsilon correction was used when the criteria for sphericity were not met. Effects sizes for main effects were calculated as partial eta squared (η_p_²) and interpreted as small (0.01), medium (0.06), and large effects (0.14) [31]. The analyses were completed with the Bonferroni post hoc test. The intraclass correlation coefficient (ICC) was calculated (95% CIs), using a two-way mixed-effect absolute agreement model [32], to verify the reliability of speed data between testers. ICC values less than 0.5 are considered poor, values between 0.5 and 0.75 are moderate, values between 0.75 and 0.9 are good, and values greater than 0.9 are excellent. Pearson’s product moment correlation test was used to determine the association between the level of the athletes and the improvement in performance. A significance level of p ≤ 0.05 was set in all analyses. The software used was SPSS (v. 25.0, IBM Corp., Chicago, IL, USA).

## RESULTS

Two athletes were excluded from the analyses for not attending all the sessions and one athlete was excluded because of foot pain before starting one experimental session. Thus, the final sample was composed of 13 power track and field athletes (sprinters, n = 6; jumpers, n = 7), whose physical and anthropometric characteristics are presented in Table 1. The sprint times evaluated by the two independent observers from 10 random recordings provided excellent reliability (ICC = 0.984, 95%CI = 0.948–0.995).

**TABLE 1:** Physical and anthropometric characteristics

Variables	Mean ± SD
Age (years)	19.0 ± 2.0
Body mass (kg)	71.7 ± 5.6
Height (cm)	177 ± 7
CMJ height (cm)	39.0 ± 3.0
Training time (y)	3.3 ± 1.1
RSI (cm/s)	3.2 ± 0.6
100-m personal best (s)	11.4 ± 0.6
200-m personal best (s)	22.7 ± 1.1
Long jump personal best (m)	5.97 ± 0.29
Triple Jump personal best (m)	13.62 ± 1.58

Legends: CMJ = countermovement jump; RSI = reactive strength index.

Two-way repeated measures of ANOVA showed that there was no effect of any factor on the overall 40-m sprint performance. There was an effect of time and condition on 0–20 m speed (Table 2). The Bonferroni post-hoc test showed that post-intervention sprint performance was worse than baseline in the DJ+DS condition (*p* = 0.02). For the 20–40-m interval, there was an effect of time on performance. Bonferroni’s post-hoc test showed that velocity in post-intervention sprint was higher compared to baseline in the DS+DJ condition (*p* = 0.015).

**FIG. 2:**
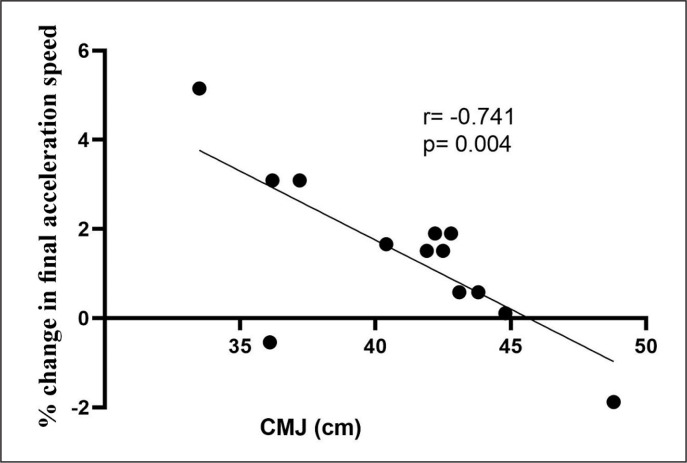
Correlation between performance improvement in the fina acceleration phase after the DS+DJ protocol and CMJ performance. DS+DJ = Dynamic stretching + Drop Jump; DJ+DS = Drop Jump + Dynamic stretching; CMJ = Countermovement Jump.

A significant relationship was found between performance in the CMJ and the percentage of change in performance in the only condition in which there was an improvement in performance from baseline (see Figure 2).

**TABLE 2:** Mean (SD) of sprint velocity at 0–20 m, 0–40 m and 20–40 m distances between conditions (Control, DS+DJ and DJ+DS) at different times (baseline and post-intervention), and statistics of the two-way repeated measures of ANOVA (n = 13)

	Control		DS+DJ		DJ + DS		Condition		Time		Condition x Time	
	Baseline	Post	Baseline	Postintervention	Baseline	Postintervention	P	η_p_²	P	η_p_²	P	η_p_²
0-20 m (m/s)	6.35 (0.24)	6.26 (0.23)	6.30 (0.27)	6.25 (0.27)	6.26 (0.25)	6.22 (0.26)*	0.014	0.299	0.022	0.366	0.289	0.098
0-40 m (m/s)	7.39 (0.27)	7.33 (0.28)	7.34 (0.30)	7.34 (0.29)	7.33 (0.28)	7.31 (0.31)	0.083	0.187	0.172	0.150	0.165	0.139
20-40 m (m/s)	8.84 (0.38)	8.83 (0.39)	8.79 (0.43)	8.91 (0.35)*	8.83 (0.35)	8.86 (0.41)	0.815	0.017	0.050	0.283	0.164	0.140

ES = Effect size; DS+DJ = Dynamic stretching + Drop Jumps; DJ+DS = Drop Jumps + Dynamic stretching;

* = p < 0.05, Bonferroni post-hoc test compared to baseline.

## DISCUSSION

The objectives of the present study were to investigate the acute effects of exercise sequence (DS+DJ vs. DJ+DS) on sprint performance and determine whether the PAPE responses are related to lower limb power. The combination of DS with DJ, independently of the sequence used, did not improve the overall performance in the 40-m sprint. However, the DS+DJ sequence promoted an increase in performance in the final acceleration phase (20–40 m). In addition, the execution of the CAs in the reverse order (i.e. DJ+DS) showed a worsening in performance in the acceleration phase. Thus, in the current study there was an indication that performing DJ after DS is effective for acute improvement of sprint performance. Although previous studies have used the combination of DS with DJ as a strategy to improve performance in the CMJ [19] and 20-m sprint [22], this is the first study comparing the influence of the order of these CAs on different sprinting phases.

The CAs used in the current study have been proven to be easy and efficient for sport settings requiring the achievement of maximum speeds after 20 m, as in the case of long and triple jumps [33]. Previously, Zimmerman et al. [34] observed an improvement in performance in 30-m sprints after a CA which consisted of continuous vertical jumps. However, no difference was found for the 10-m sprint performance. In contrast to our results, Byrne et al. [22] and Turki et al. [17] previously found performance improvements in the 20-m sprints after using DS combined with depth jumps and DS alone in team sport athletes. These discrepancies between studies may be explained by differences in samples’ characteristics, CAs used and sprinting distances evaluated. While team sport players commonly execute sprints at or near to maximum velocities over distances ≤ 20-m during sport-specific activities [35], for track and field sprinters, these distances are typically used to develop their initial acceleration abilities [36]. Further studies are warranted to determine the relative influence of these factors on sprint performances in different sport settings.

We selected two exercises commonly used in training and competitive settings [12]. Previous evidence has demonstrated that plyometric jumps primarily recruit type II fibres, thus favouring the potentiation mechanisms [9] which are related to performance improvements in speed and strength tests [13]. However, DS may decrease the time to peak torque and increase the rate of torque development [37]. We hypothesized that the combination of these stimuli could increase performance after both protocols. However, we identified no performance gains in the acceleration phase for the DS+DJ condition, perhaps because this phase requires greater activation of the leg and hip extensor muscles [38], with a more horizontal body position at the beginning of the phase [36], thus differing from the muscles and body position used in the DJs. The performance improvement in the final acceleration phase can be characterized by shorter contact times and a more vertical position of the body [36], which is more related to the execution of DJs. While these arguments are speculative, further studies should better explore the influence of the neuromuscular, kinetic and kinematic factors involved on these acute responses after exercises with different biomechanical characteristics [39], as it would seem that the change of the order between DJs and DS may influence these acute responses.

The negative correlation found between jump capacity and PAPE in final acceleration speed may indicate a greater potentiating effect in athletes with a lower level of lower limb power (see Figure 2). This is somewhat contrary to the suggestions by Wilson et al. [2], who were the first to systematically demonstrate an association between training experience and PAPE responses. In this regard, Boullosa [26] recently suggested that the two main PAPE moderating factors related to the population of athletes are strength levels and training experience; however, the consideration of other potential moderators such as sex and age has been poorly addressed in applied studies. Furthermore, previous studies indicating an influence of training background and strength levels have been conducted with traditional strength training exercises [2, 40]. Therefore, as the athletes in the current study are young power track and field athletes with limited training experience, and because we used a novel combination of ballistics exercises, the combination of these factors would explain this divergence from the current evidence. Further studies should investigate in more detail the influence of population characteristics in the PAPE response with different exercises.

Considering the performance improvement in the 20–40-m section**,** we may suggest the use of this PAPE strategy for long jumpers, because the approach runs are 35–40 m and thus a higher final acceleration speed could be achieved at the end of the approaching run, therefore potentially increasing the long jump distance [41]. Similarly, sprinters may use this strategy because we would expect that this effect could be maintained during the maximum speed phase of a 100-m run (50–70 m), which is a key phase for performance. However, these potential benefits should be tested in future interventions in both athletic populations.

## CONCLUSIONS

In conclusion, using the combination of DS with DJ, independently of the sequence used, did not improve overall performance in the 40-m sprint. The DS+DJ sequence improved performance in the final acceleration phase (20–40 m). Athletes with lower levels of lower limb power may benefit more from this CA. When DS is preceded by DJ, there was a worsening in the initial acceleration. These findings provide coaches with a simple and practical PAPE strategy to favour better sprinting performances without the use of additional equipment and complex settings.
